# On the use of AI for Generation of Functional Music to Improve Mental Health

**DOI:** 10.3389/frai.2020.497864

**Published:** 2020-11-19

**Authors:** Duncan Williams, Victoria J. Hodge, Chia-Yu Wu

**Affiliations:** ^1^ Digital Creativity Labs, University of York, York, United Kingdom; ^2^ Department of Computer Science, University of York, York, United Kingdom; ^3^ School of Science, Engineering and Environment, University of Salford, York, United Kingdom; ^4^ Department of Electronic Engineering, University of York, York, United Kingdom

**Keywords:** mental health, emotional states, feedback, biophysiological sensors, generative music, machine learning, artificial intelligence, algorithmic composition

## Abstract

Increasingly music has been shown to have both physical and mental health benefits including improvements in cardiovascular health, a link to reduction of cases of dementia in elderly populations, and improvements in markers of general mental well-being such as stress reduction. Here, we describe short case studies addressing general mental well-being (anxiety, stress-reduction) through AI-driven music generation. Engaging in active listening and music-making activities (especially for at risk age groups) can be particularly beneficial, and the practice of music therapy has been shown to be helpful in a range of use cases across a wide age range. However, access to music-making can be prohibitive in terms of access to expertize, materials, and cost. Furthermore the use of existing music for functional outcomes (such as targeted improvement in physical and mental health markers suggested above) can be hindered by issues of repetition and subsequent over-familiarity with existing material. In this paper, we describe machine learning approaches which create functional music informed by biophysiological measurement across two case studies, with target emotional states at opposing ends of a Cartesian affective space (a dimensional emotion space with points ranging from descriptors from relaxation, to fear). Galvanic skin response is used as a marker of psychological arousal and as an estimate of emotional state to be used as a control signal in the training of the machine learning algorithm. This algorithm creates a non-linear time series of musical features for sound synthesis “on-the-fly”, using a perceptually informed musical feature similarity model. We find an interaction between familiarity and perceived emotional response. We also report on subsequent psychometric evaluation of the generated material, and consider how these - and similar techniques - might be useful for a range of functional music generation tasks, for example, in nonlinear sound-tracking such as that found in interactive media or video games.

## Introduction

There is increasing evidence that mindfulness can form a positive contributor to mental health and general wellbeing (Baker and Bor, 2008; Economides et al., 2018). In this work we describe the design and evaluation of a system combining machine learning (ML) approaches with biophysiological metering and psychological evaluation of two descriptors which we consider to be at discrete ends of an affective space with positive mental health states at one side of the space (mindfulness, calmness, etc.), and negative mental states at the other side of the space (fear, anger, etc.) (Chambers et al., 2009).

The distinction between affective state, emotion, and mood, is complex, and is generally drawn between the duration of the response (Calvo et al., 2009). Various models of affective state exist, including models with dimensions for positivity and activation strength, such as the cirumplex model of affect (Russell, 1980). This model places valence (as a measure of positivity) and arousal (as a measure of activation strength) on the horizontal and vertical axes respectively. Emotional descriptors (e.g., happy, sad, angry) can be mapped on to this space. Other models exist, for example, multidimensional models which also include, for example, dimensions for dominance. This type of model might be useful when delineating between very intense and very negative emotional descriptors, such as the difference between anger and fear–both intense, and negative, but one being a more dominant response and the other more passive. Often, individual emotional descriptors can be plotted across these types of spaces (Williams et al., 2014). In the case of this work, we consider a general model with two specific descriptors as approximately at either end of a scale–mindful, and afraid. However, the descriptors themselves are open to debate and could certainly form the subject of further work. We intend to explore the use of AI to generate music intended to elicit differing emotional states in an abstract emotional space and to examine biophysiological markers in a synchronous manner.

Existing work has shown that there are responses to music in both the central and peripheral nervous system (in other words, both physiological, and neurological responses) (Aldridge, 2005; Calvo et al., 2009). When listening to enjoyable music, the listeners pupils may dilate, or they might experience a change in heart rate, blood pressure, and skin conductivity (Blood et al., 1999; Daly et al., 2015). Measurement of galvanic skin response (GSR) has been shown to be a robust metric for analysis of emotional responses to music (Shrift, 1954; Vanderark and Ely, 1993; Daly et al., 2015).

Thus, there is a potential crossover between mental state, physiological reaction, and musical stimulation. Chambers (Chambers et al., 2009) showed that states of mindfulness have correlations in GSR (otherwise known as electrodermal activity, or skin conductivity), heart rate variability, and the ratio of alpha and beta waves in electroencephalographic measurement. The electroencephalograph (EEG) is a technique for metering electrical activity from the scalp used to infer patterns of brain activity. Bondolfi (Bondolfi, 2013) and Economides et al. (Economides et al., 2018) proposed that proactive training and entrainment of mental states might thus contribute to therapeutic treatment and physiological improvement.

We aim to harness these findings to create a machine learning based music-training system to encourage a change in affective state as measured through biophysiological correlations. For example, mood-based regulation (becoming less afraid or anxious) might be a useful mental health target for the user. Beyond mental health this type of system could have applications in the creative industries, for example, in film, television, or video games (Knox et al., 2008; Williams et al., 2015a), in which case, the viewer or player might be subjected to targeted mood disruption (i.e., rather than being calmed by the musical stimulus, the listener might preferably be deliberately excited, or even scared in the case of some types of gameplay).

In this paper we draw on previous experimental work documented in (Williams et al., 2019a; Williams et al., 2019b), and more widely, machine learning, a field of computer science covering systems that learn “*when they change their behavior in a way that makes them perform better in the future*” (Witten et al., 2016). These systems learn from data without being specifically programmed. Kim et al. (Kim et al., 2010) and Laurier and Herrera (Laurier and Herrera, 2012) give a literature overview of detecting emotion in music and focus on the music representations. Laurier and Herrera also provide an analysis of the machine learning algorithms used by systems in their survey. Classification algorithms used in the literature include C4.5, Gaussian mixture models, k-nearest neighbor, random forest, support vector machines, (Kim et al., 2010; Laurier and Herrera, 2012; Mostafavi et al., 2013). Regression techniques include Gaussian mixture model regression, multiple linear regression, partial least-squares regression and support vector regression.

ML has been used to retrieve music by mood and ML analyses found the personalized approach more consistent than a general approach (Mostafavi et al., 2013). An example is supervised learning. In supervised learning, the algorithm learns from a set of labeled inputs. It then generates a model associating the inputs with their respective labels or scores, and then classifies (or predicts) the likely label for previously unseen examples using the learned model. We use supervised learning to label newly generated material with potential emotional descriptors in the work documented in this paper.

Real-world testing of systems using bio-signal mappings in music generation contexts has become an emerging field of research, partly due to recent advances in portability, wearability, and affordability of biosensors. For example, Huang and Cai (Huang and Cai, 2017) generate simple music for specific emotional states using Markov chains. The Markov chains are used to generate music while the user wears a heart-rate sensor to monitor their bio-physiological response to the created music. The system was able to generate emotionally responsive music in a limited trial considering basic emotional descriptors. We have developed another such system, which assumes lower skin conduction variability as a correlate for positive affective state. It attempts to generate emotionally congruent music as a training tool to promote positive affective states in the context of mindfulness. In the future, this system could also work in reverse by using skin conductance variability as a control signal to inform musical feature mapping for non-linear music generation.

While the physical and mental health benefits of music use have increasingly been reported upon (including improvements in cardiovascular health (Szmedra and Bacharach, 1998)), reduction of dementia in elderly populations (Vink et al., 2003), stress reduction (Knight and Rickard, 2001) and so on), the use of existing music to target such outcomes can be problematic due to the influence of familiarity, or repetition of stimulus materials (Kim, 2011; Ladinig and Schellenberg, 2012). Thus a major focus of this work is to evaluate a system for the automatic generation of new musical materials with functional aims (improvement of listener affective state on a case-by-case basis as determined by self-report or biophysiological correlate). We therefore aim to:

(1) Measure musical features according to a similarity model from the human-labeled dataset and use these to inform Markov-model generation of new music to be evaluated by a supervised learning algorithm
(2) Evaluate the success of the supervised learning algorithm using self-report and biophysiological measurement of GSR

We hypothesize that music generated by the automated algorithm may be able to influence self-reported emotional state and GSR, when compared to music which listeners may already be familiar with from a corpus of popular film music.

## Materials and Methods

GSR is used as a marker of psychological arousal and as an estimate of emotional state to be used as a control signal in the training of the ML algorithm. This algorithm creates a non-linear time series of musical features for sound synthesis “on-the-fly,” using a perceptually informed musical feature similarity model.

We use the system described in (Williams et al., 2017) to create functional music informed by biophysiological measurement across two case studies, with target emotional states at opposing ends of a Cartesian affective space (a dimensional emotion space with points ranging from descriptors from relaxation, to fear).

**FIGURE 1:**
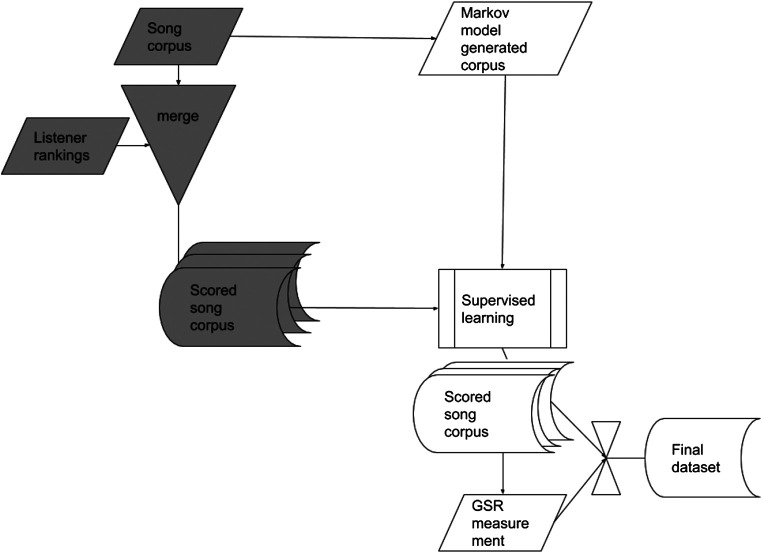
Listeners rank musical excerpts to produce labeled excerpts (this process is shown in gray). These excerpts are analyzed for features to train a supervised learning model for construction of new excerpts. New excerpts are evaluated by means of GSR and self-report feedback to revise the scores according to sensor results and produce our adaptive system and music excerpts dataset. Shading indicates different stages of process.

The system detects the user’s current emotional level and the ML algorithm picks musical pieces to influence their future emotional level to achieve their desired mood. This whole process requires musical pieces that have an associated emotional label (score) to allow the selection of appropriate pieces. We use two tasks to achieve this, illustrated in Figure 1. Firstly we analyze a corpus for musical features using classification and regression. We use a multi-feature music representation combining analysis of symbolic musical feature data from a MIDI file, which represents the structure of the melody, chords, rhythm and other musical properties concerning timing, dynamics, and pitch, with Mel-Frequency Cepstral Coefficients features (Logan, 2000) obtained from the entire piece to represent the timbral quality of the instrumentation. This dual representation is more flexible and richer than either MIDI or signal-based audio content features alone. We only use numerical data features to describe each piece and perform feature selection to identify the most significant set of features as described in (Hodge et al., 2016). Using this reduced set of significant features, the ML model can predict the likely emotional state score that a human listener would ascribe to newly input music pieces by determining the similarity between pieces using their respective sets of features.

We train the supervised learning algorithm to expand on a human-labeled corpus, which was labeled by means of a survey of 53 participants using a Qualtrics on-line survey (www.qulatrics.com). Each participant evaluated four musical excerpts, two calm or positive (N1 and N2) and two anxious or negative excerpts (S1 and S2), in a bipolar ranking across six pairs choosing the positive in each pair {N1vsS1, S2vsN2, S1vsN2, N1vsS2, S1vsS2 and N1vsN2}. The survey presented an initial question to allow the user to familiarize themselves with the format and then presented the six questions. The Qualtrics questionnaire allowed us to specify that each track played in full to each participant to ensure that the participant adapted fully to the track. We randomized the order of presentation of the questions (pairs of tracks) to each of the participants to reduce contextual effects. Participants were not required to answer every question in order to complete the evaluation. The algorithmic composition system uses hidden Markov models (HMM) to create new music to provide sufficient quantities of labeled pieces for the system to operate. We use a transformative algorithm based on a second order Markov-model with a musical feature matrix. New material is formed of permutations of the HMM with deliberate feature constraints following the procedure described in (Williams et al., 2015b; Williams et al., 2017). This allows discrete control over five parameters in a 2-dimensional model.

Human experiments are only feasible on a small set of music pieces as *n* pieces of music require *n!* comparisons and enough human survey participants to provide enough responses for each of the *n!* comparisons. Using human participants to generate a sufficiently large database of labeled pieces for this work would be very time consuming and complex. From our Qualtrics analyses we were able to train our ML model using supervised learning to map the musical sequences to scores where the sequences are represented by features as described previously. Our generative system can be used to create new musical sequences according to the likelihood of a particular affective state occurring after the current and preceding states measured in the listener and these can be scored by the trained ML model.

We then analyze the listener’s GSR to select music which exercises the most influence of the listener’s affective state. We incorporate a feedback loop to adapt the corpus scores according to the user’s affective response, selecting musically consistent pieces and removing pieces that do not influence the user’s emotional level (in essence a fitness function). We then compare the listener’s GSR signal, the emotional tag they describe after listening and the calmness level of the piece the participant is listening to. To analyze GSR, we used the Shimmer3 wireless GSR + Unit^1^ which has been validated for use in biomedical-oriented research applications. This device needs to be calibrated on each use to establish a baseline skin conductance signal, which varies due to many factors including skin dryness, nervousness (due to unfamiliarity with the experimental procedure) and ambient temperature. The captured reading for each user under analysis is their skin conductance response while undertaking the listening exercise, minus their individual skin conductance response baseline. After listening to each piece, the users completed a questionnaire describing the emotion they felt while listening which we compared to the GSR data.

## Results

Responses to the musical stimuli suggest that listeners found it relatively easy to discriminate the affective states between stimuli, which were rendered using different synthesized timbres. Generally speaking, shorter durations and larger pitch ranges were considered lower in positivity (for example, “more anxious”) than longer durations with a more restricted pitch range, regardless of the musical timbres. 58.1% of participants thought S2 was more negative than S1 while 54.6% felt N1 was more negative than N2.

For S1, 94.5 and 93.2% of participants rated it more negatively than N1 and N2 respectively. For S2, 88.1 and 89.1% of participants rated it more negatively than N1 and N2 respectively. Yet, 58.1% of the participants rated S2 more negatively than S1 despite S2 having created more positive report than S1 when compared to the positive stimuli. Similarly, for N1, 4.6 and 10.9% rated it more negatively than S1 and S2, respectively, while for N2, 6.8 and 11.9% rated it more negatively than S1 and S2, respectively. This presents a similar contradiction as for the negative stimuli as N1 has lowest reported positivity yet was rated more negatively than N2 by 54.6% of participants.

From these comparisons, we were able to attain sufficient data that we can calculate a ranked order (score) for the pieces from these pairwise comparisons (Wauthier et al., 2013). In order for the biofeedback based evaluation to be feasible, we then use the supervised learning generated corpus to provide a large enough quantity of stimuli.

Our analyses revealed that there is a direct correlation between the reported negativity of a musical piece, the user’s GSR readings and the emotions they describe feeling in a questionnaire survey conducted after listening. Users display elevated GSR for negative pieces which they also labeled congruently in the questionnaire and lower GSR and appropriate labels for calmer pieces. We also find an interaction between familiarity of existing material in the corpus, and the perceived emotional response.

## Discussion

Our experiments highlighted that familiarity influences individual affective responses both in self-report and in GSR. For this reason, we have attempted to focus on generating novel music to create functional music which responds to a listener’s biophysiological state rather than invoked or evoking memories (and removing some need to consider the influence of familiarity might affect listener responses).

Overall, we saw an increased GSR in each music excerpt, regardless of whether the excerpts were generated by the HMM model or not. We conclude that GSR is a suitable detection tool to evaluate emotional responses. DES-based self-report was used to allow listeners to report on different categories of emotions (Lane et al., 1990). However, the two measurements do not have consistent results when considered in response to music that listeners described themselves as being familiar with (samples of famous film music). Nevertheless, the emotional responses to generated music excerpt *g1* showed consistent results with both self-reporting and GSR. Thus we consider there may be an interaction between music and familiarity (perceived emotions). In self-reports, familiarity has insignificant effects. Conversely, in GSR data, there are differences in the simple effect of music between unfamiliar and familiar tracks. Familiar movie soundtracks also have higher GSR amplitude than unfamiliar ones but lower negative self-reports.

Hence, to induce calm states of mind reliably we believe further work should focus on unfamiliar music composed using artificial intelligence based approaches. Our main aim for this work is to develop a music generator for music therapy use that produces music which induces specific emotions in the listener but the approach described here might also be suitable in the design of a more generic music generator capable of inducing specific emotions in the audience, specifically when functional music with non-linear duration would be useful (e.g., videogame sound-tracking and the creative industries more broadly construed).

## Conclusions and Further Work

This work suggests that generative music technology has the potential to produce infinite soundtracks in sympathy with a listener’s bio-signals, and in a biofeedback loop. There are promising applications for linking music with emotions, especially in the creative industries art and therapy, and particularly for relaxation. Enhancement of well-being using music and the emotions music induces is becoming an emerging topic for further work. We have applied a system for musical feature analysis from MIDI features and Mel-Frequency Cepstral Coefficients features (Logan, 2000) to train a supervised learning algorithm with listener responses to a corpus of training material. We use this algorithm to influence the generation of a larger corpus by means of a Hidden Markov Model algorithmic composition engine, and then analyzed the complete corpus by testing listener GSR and self-report in a DES evaluation. GSR is used as a marker of psychological arousal and as an estimate of emotional state to be used as a control signal in the training of the ML algorithm. This algorithm creates a non-linear time series of musical features for sound synthesis “on-the-fly”, using a perceptually informed musical feature similarity model. These small case studies, with target emotional states at opposing ends of a Cartesian affective space (a dimensional emotion space with points ranging from descriptors from positive descriptor states such as calmness, to negative descriptors such as fear), show us an interaction between familiarity and perceived emotional response. We believe further work involves three major challenges.

(1) The extraction of meaningful control information from signals emanating from the body.
(2) Design of generative and performative music technology in order to respond to such information.
(3) Consideration of the ways in which such technology can be best deployed depending on the intended end-use; for example, in therapeutic contexts.

There is a tendency in human-computer interaction work for music generation to prioritize the technical implementation by focusing on increased speed or accuracy of a system, rather than the specific needs of the application. In a music therapy context, for example, one advantage of a functional music system is that it might be used by a patient with no musical ability and thereby potentially increases their own ability to express emotional states and have access to the pleasure of performing music with other people. Thus the use of biophysiological sensors is critical in the development of suitable systems for audio generation in the context of mindfulness or relaxation where improved affective state as part of mental health is an intended outcome. These are not trivial considerations in terms of application design, and subsequent evaluation. Methodologies for evaluating the success or failure of such systems remain a significant challenge for further work.

## Data Availability Statement

The datasets analyzed in this article are not publicly available. Requests to access the datasets should be directed to d.a.h.williams@salford.ac.uk.

## Ethics Statement

The experiment was conducted with ethical approval from the University of York, Dept of Electronic Engineering review board. The patients/participants provided their written informed consent to participate in this study.

## Author Contributions

DW and VH contributed conception and design of the study; DW composed the musical sequence database; CW led the human analyses under guidance from DW and VH, VH developed the Qualtrics survey, CW and VH performed the statistical analyses; DW wrote the first draft of the article; VH and CW wrote sections of the article. All authors contributed to article revision, read and approved the submitted version.

## Conflict of Interest

The authors declare that the research was conducted in the absence of any commercial or financial relationships that could be construed as a potential conflict of interest.

## Footnotes

1
http://www.shimmersensing.com/products/shimmer3-wireless-GSRsensor
